# A characterization study on electroencephalographic changes and lateralization of functional brain connectivity in boys with developmental coordination disorder

**DOI:** 10.3389/fneur.2025.1591236

**Published:** 2025-10-29

**Authors:** Yiqi Song, Zhenpeng Li, Xiaotong Zhu, Guang Pei, Juanyi Hou, Shiji Liu, Yi Xie, Yongtao Xie

**Affiliations:** ^1^College of Physical Education and Sports, Beijing Normal University, Beijing, China; ^2^Department of Physical Education, North China University of Science and Technology, Sanhe, China; ^3^Hebei University of Water Resources and Electric Engineering, Cangzhou, China; ^4^Tianjin Normal University, Tianjin, China; ^5^Outpatient Department, The 980th Hospital of the Joint Logistics Support Force, Shijiazhuang, China; ^6^Hebei Sport University, Shijiazhuang, China

**Keywords:** electroencephalographic, DCD, boys, motor skill, functional connectivity

## Abstract

**Objective:**

Children’s motor development is closely related to the development of their brain functions. Currently, the central neural mechanisms in children with developmental coordination disorder (DCD) are poorly understood. This study investigated the changes of EEG patterns in DCD boys.

**Methods:**

In this study, 15 boys with DCD were screened via the Movement Assessment Battery for Children 2nd Edition (MABC-2), and 15 boys with typical development (TD) at the same age were matched as the control group. The electroencephalographic (EEG) signals of the boys were recorded in the resting state and during the visual motor integration (VMI) task, and the relative power, sample entropy (SampEn), phase lag index (PLI), and lateralization of functional connectivity were analyzed.

**Results:**

In the resting state, no abnormal changes were found in the relative power of the EEG or SampEn of the DCD boys (*p* > 0.05), and the PLI of each frequency band in the DCD boys was significantly lower than that in the TD boys (*p* < 0.001). During the VMI task, the *θ* power of the DCD boys decreased significantly at the right frontal central border (FC2: *p* < 0.05), the *β* power decreased significantly at the right frontal central border (FC2: *p* < 0.001), and the midline of the parietal region (Pz: *p* < 0.001), and there was no abnormal change in SampEn. The PLIs of the *α*, *β*, and *γ* frequency bands in DCD boys were significantly lower than those in TD boys (*p* < 0.001), and the functional connectivity of the *β* band around the cerebral motor cortex was significantly lateralized right hemispheric acceleration (*p* < 0.05).

**Conclusion:**

The brain functional network connectivity of DCD boys may have developmental defects, and the abnormal changes in brain activation, functional connectivity, and lateralization of functional connectivity during movement may be important brain mechanisms for their poor motor coordination. These findings provide a new perspective for analyzing and evaluating the brain function of DCD children.

## 1 Introduction

With the development of society, children’s neurodevelopmental issues, such as attention deficit hyperactivity disorder (ADHD) and autism spectrum disorder (ASD), are receiving increasing attention. Children with poor motor development belong to the category of developmental disorders, and it is believed that children with poor development of fine motor skills may develop developmental coordination disorder (DCD) in the future (1). DCD is a neurodevelopmental disorder (2) that is characterized by a marked impairment in motor skills in terms of coordination and severe interference with daily life. The prevalence of DCD in school-age children ranges from 1.7 to 6%, with a ratio of 4 ~ 7:1 between boys and girls, and DCD can persist into adulthood (3). The pathogenesis of DCD has been studied and analyzed in several studies; however, the pathogenesis of DCD is still unclear. DCD has various manifestations of movement disorders, such as dystonia, lack of stability of movement, and movement control disorders (4, 5). Analysis suggests that these manifestations may be related to issues such as the execution of motor tasks, proprioception, sensory integration, and the processing of visual information (6). The above viewpoints may be the central mechanisms underlying delayed motor performance in children with DCD, but they cannot fully represent the causes of DCD. Given the recognized link between motor difficulties and brain features, few studies have systematically explored the brains of DCD children (7). Therefore, it is necessary to study the central mechanism of motor developmental delay in DCD children from the perspective of brain function.

Kaplan et al. (8) suggested that DCD may be due to “atypical brain development”. One study reported that neuroelectrophysiological methods (EEGs) can provide insights into atypical motor development (9). Meachon et al. (10) studied the resting-state EEG power spectra of 12 adults with DCD. They reported that in the eyes-closed resting state, frontal beta power and occipital high gamma power were significantly different. de Castelnau et al. (11) reported that there was a significant increase in connectivity between frontal center regions of the brain of DCD patients in the state of performing an action task. In contrast, McLeod et al. reported that, compared with normal children, children with DCD presented reduced connectivity between several brain regions, as analyzed by resting-state fMRI (12). In addition, a study revealed that the left motor cortex activation pattern in resting children with DCD was similar to that in younger children, suggesting that there is a “maturational lag” in the brain activation of motor functions in children with DCD (13). Although some studies have been conducted on DCD children via EEG methods, the number of related studies is relatively limited, and these studies also have common problems, such as inconsistent research status of DCD patients and incomplete analysis of EEG data under the same state. To gain a deeper understanding of the changes in the central nervous system of DCD children, this study will conduct a comprehensive analysis of EEG data of DCD children under resting state and action task conditions using multiple indicators, fully exploring the characteristics of EEG data changes in DCD children to reveal the brain mechanisms of DCD children.

## 2 Methods

### 2.1 The subject of the study

Boys aged 6–8 years in a general public elementary school in a city in Hebei Province, China, were selected as screening subjects. Fifteen boys with poor coordinated development were included in the study by evaluating the results of the MABC-2. Moreover, 15 boys with typical development matched by age and sex were included as a control group. The subjects of this study were divided into two groups, DCD boys and TD boys, all of whom were right-handed. The general demographic data of the two groups are shown in Table 1. This study was approved by the ethics committee, and the guardians of the children involved signed informed consent forms. Study was conducted between September and October of 2024.

**Table 1:** Demographic information of the subjects.

Groups	*n*	Dominant hand	Age	*Z*	*p*
DCD	15	Right	7.21 ± 0.71	−0.114	0.910
TD	15	Right	7.18 ± 0.71		

### 2.2 MABC-2 and motor tasks

**Figure 1:**
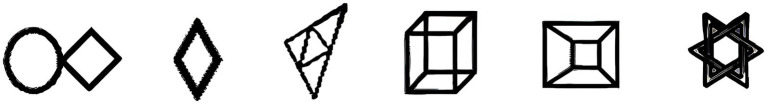
Illustration of VMI tasks.

(1) The MABC-2 was used to test the motor coordination ability of the study participants. The MABC-2 has demonstrated good internal consistency and test–retest reliability. The assessment consists of eight tasks organized into three subsets: manual dexterity, aiming and catching, and static and dynamic balance. The MABC-2 has demonstrated good internal consistency and test–retest reliability. The assessment consists of eight tasks organized into three subsets: manual dexterity, aiming and catching, and static and dynamic balance. Age-adjusted standard scores are provided for each subtest, and a total score from which percentiles can be derived (14); in our study, a total score below the 25th percentile (standard score <9) indicates developmental coordination disorder.
(2) Motor tasks: Studies have revealed a positive correlation between DCD children’s ability to reproduce depictions and their MABC scores (15). The design of the motor task during EEG acquisition in the present study was based on some of the graphs in the Berry–Buktenica development-mental test of visual-motor integration (VMI), which were duplicated and combined into 18 geometric shapes, the graphic forms of which are shown in Figure 1. The participants were asked to reproduce the depictions by directly copying the visual models presented to them.

### 2.3 EEG data acquisition and preprocessing

**Figure 2:**
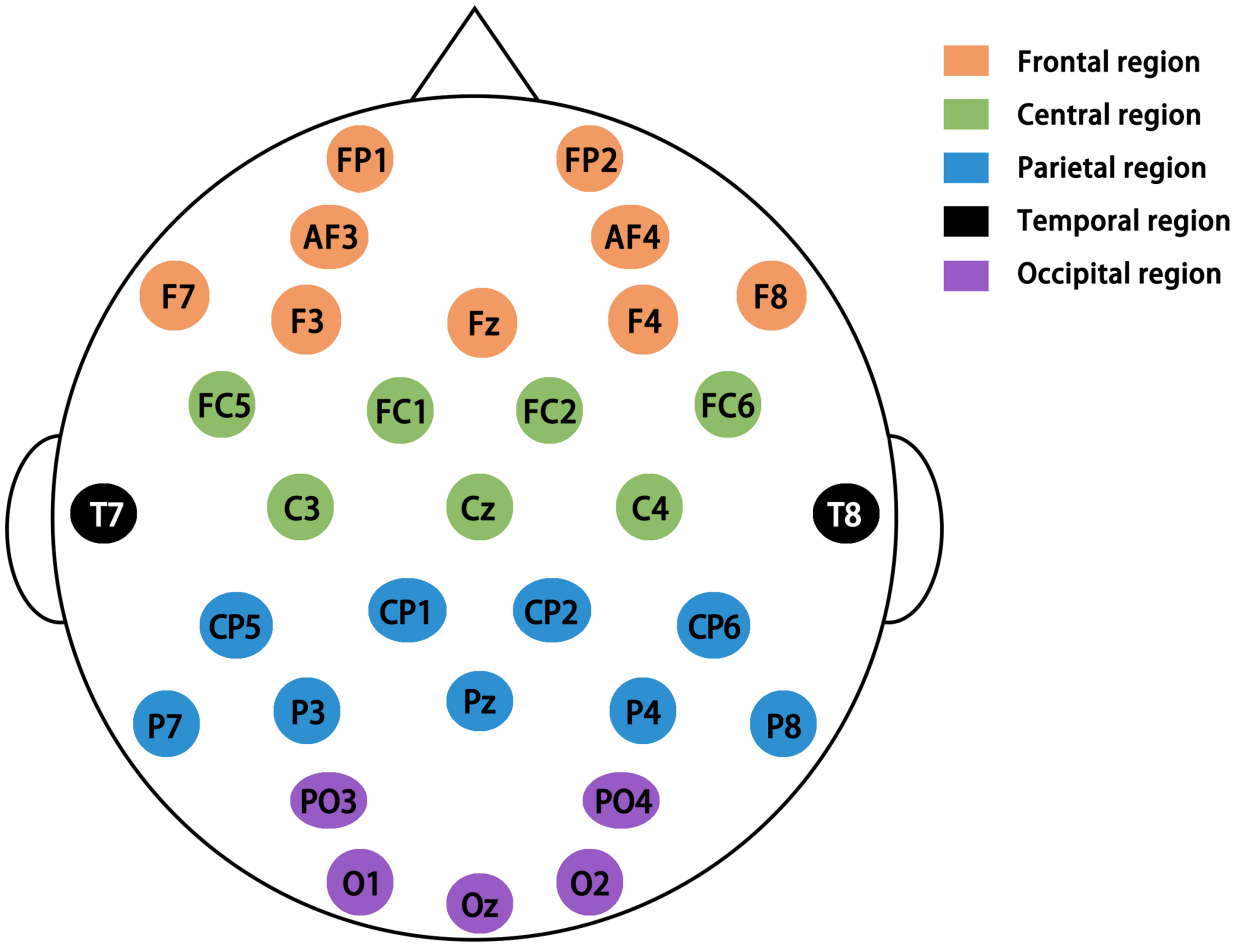
Diagram showing electrode positions for the 32-channel International 10–20 system (45).

EEG data were collected via a 32-channel Enobio wearable EEG system, with 32-channel electrodes arranged according to the international standard 10–20 system (Figure 2) and a sampling frequency of 500 Hz. Acquisition procedure: The subjects sat still for 5 min after entering the laboratory, put on the EEG cap, adjusted the equipment (resistance <5 kΩ), and rested with their eyes open for 3 min in a comfortable sitting position, while resting EEG data were collected. The resting EEG data were collected at the same time. After the resting EEG data were collected, the EEG data were collected when the subjects completed the graphic tracing task.

The original EEG time series data were preprocessed, and the specific steps included localization, referencing, filtering, deleting or differential bad channel data, removing artifacts and segmentation, etc. The preprocessed data were analyzed and processed in the next step. In this study, the cleaned EEG data were then filtered into five frequency bands (*δ*: 0.5–4 Hz; *θ*: 4–8 Hz; *α*: 8–13 Hz; *β*: 13–30 Hz; *γ*: 30–45 Hz) (16).

### 2.4 Calculation of EEG data indicators

The main analytical methods for EEG signal feature extraction in this study are frequency domain analysis (power spectrum estimation, PSD), nonlinear signal complexity analysis (sample entropy, SampEn), and functional brain network connectivity analysis (phase lag index, PLI).

(1) PSD

By using a Fourier transform to convert EEG signals into the corresponding relationship between frequency and power, an improved period Gram method called the Welch method is used to estimate the power spectrum of EEG data in different frequency bands. The absolute PSD of the corresponding frequency bands (*δ*, *θ*, *α*, *β*, *γ*) is then extracted from the spectrum, and the relative power is calculated through the absolute PSD of each frequency band.

(2) SampEn

By measuring the probability of generating a new pattern in the signal, SampEn is a measure of the complexity of a time series; the greater the probability of generating a new pattern is, the greater the complexity of the series. The formula is:


$$\text{SampEn}(m,r)=-\ln\;[\frac{A^{m}(r)}{B^{m}(r)}]$$

On the basis of experience, *m* was taken as 2, and *r* was taken as 0.2 SD for calculating SampEn. A: the number of pairs of length *m* + 1 vectors whose distance is less than or equal to the tolerance *r*, excluding self-matches. B: the number of pairs of length *m* vectors whose distance is less than or equal to the tolerance *r*, excluding self-matches. The specific calculation process and parameter meanings can refer to the literature of Alcaraz et al. (17).

(3) PLI and functional connectivity lateralization

Functional brain networks are composed of nodes and edges formed by the connecting lines between nodes. In the functional brain network model, nodes represent brain regions, and edges reflect the functional and effective connections between different brain regions. In this study, the electrodes corresponding to the EEG signals are defined as nodes, the dependencies between nodes are defined as edges, and synchronization is used as the carrier of the dependencies. Since the PLI can avoid the volumetric conduction effect in the brain, the PLI was chosen as the index to reflect the synchronization between nodes in this study. The calculation procedure of this index can be found in Lustenberger et al. (18).

Asymmetry in brain structure and function between the left and right hemispheres, known as cerebral lateralization, is a well-established phenomenon. The functional asymmetry between the left and right hemispheres of the brain is an important feature of the functional organization of the brain, and the coordinating function of the brain in human locomotion is characterized by lateralization (19). In this context, the present study analyzed the functional connectivity lateralization index in children. According to this point of view, the brain functional connectivity lateralization index of children with DCD was analyzed in this study. Based on the significant group differences in phase lag index (PLI) observed during the VMI condition, we further assessed functional connectivity lateralization between the two groups. The procedures were as follows: (1) Selection of electrode pairs: The screening criteria are that any pair of electrodes cannot be located in two hemispheres of the brain or have midline electrodes. According to this standard, one pair, six pairs, and seven pairs of electrodes with significant differences were selected for lateralization calculation in the alpha, beta, and gamma frequency bands, respectively. (2) Contralateral matching: For each selected electrode pair in one hemisphere, we identified a corresponding pair in the contralateral hemisphere representing the same brain regions. For example, the pair F8–P4 in the right hemisphere was matched with F7–P3 in the left hemisphere. (3) Computation of lateralization index: The lateralization index, or asymmetry score (AS), was calculated using the following formula: AS(*x*) = (*x*(l)−*x*(*r*))/(*x*(l) + *x*(*r*)), where *x*(l) represents the value of the left brain index and *x*(*r*) represents the value of the right brain index. For example: AS = (PLI(F7–P3) – PLI(F8–P4)) / (PLI(F7–P3) + PLI(F8–P4)). A positive value of AS represents the lateralization of the functional connectivity of the left brain, and a negative value of AS represents the lateralization of the functional connectivity of the right brain.

### 2.5 Data analyses

For the statistical analysis, SPSS 23.0 software was used to perform independent samples *t*-tests or Mann–Whitney *U*-tests for demographic data and motor coordination test scores, respectively; *p* < 0.05 was used as the criterion for a statistically significant difference. MATLAB software was used to compare the EEG indices, the Mann–Whitney *U*-test was used to compare the means of each index, and the false discovery rate (FDR) method was used to correct for multiple comparisons, with the threshold set at *p* < 0.05.

## 3 Results

### 3.1 Comparison of MABC-2 scores between groups

In terms of the MABC-2 scores, the boys in the DCD group lagged behind the boys in the control group (TD boys) in terms of the MABC total score (TD = 10.25 ± 1.21, DCD = 5.67 ± 1.67; *p* = 0.000), manual dexterity (TD = 10.08 ± 1.56, DCD = 7.25 ± 1.86; *p* = 0.001), aiming and catching (TD = 10.42 ± 2.07, DCD = 4.42 ± 2.43; *p* = 0.000), and static and dynamic balance (TD = 9.67 ± 0.98, DCD: 7.17 ± 2.73; *p* = 0.010). The results are shown in Table 2, which reveals that there is a significant difference between the two groups of boys in the development of their motor skills.

**Table 2:** Differences in MABC-2 scores between DCD and TD boys.

Index	TD (*n* = 15)	DCD (*n* = 15)	*t*	*p*
Manual dexterity	10.08 ± 1.56	7.25 ± 1.86^**^	4.032	0.001
Aiming and catching	10.42 ± 2.07	4.42 ± 2.43^***^	6.519	0.000
Static and dynamic balance	9.67 ± 0.98	7.17 ± 2.73^*^	2.989	0.010
MABC-2 total score	10.25 ± 1.21	5.67 ± 1.67^***^	7.688	0.000

^*^p < 0.05; ^**^p < 0.01; ^***^p < 0.001.

### 3.2 Comparison of the resting-state data between the groups

(1) Comparison of PSD

No statistically significant difference in resting-state relative power was found between the DCD boys and the TD boys (*p* > 0.05).

(2) Comparison of SampEn

No statistically significant difference was found between the resting EEG SampEn values of the DCD boys and those of the TD boys (*p* > 0.05). The results suggested that the EEG signal complexity of the DCD boys was normal under resting conditions.

(3) Comparison of PLI values

PLI is a measure of the phase coherence between the EEG signals of two electrodes at a certain frequency, and a high degree of coherence indicates a high level of functional brain connectivity and a high level of functional integration between neuronal populations, and vice versa, suggesting that neuronal populations are functionally separate. The resting-state PLIs of the DCD boys were significantly lower than those of the TD boys in all frequency bands (*p* < 0.001). The results are shown in Table 3.

**Table 3:** Differences in the resting-state PLIs between DCD and TD boys.

Band	TD (*n* = 15)	DCD (*n* = 15)	*t*	*p*
All band	0.319 ± 0.005	0.209 ± 0.002^***^	−114.041	0.000
Delta	0.521 ± 0.003	0.371 ± 0.002^***^	−269.625	0.000
Theta	0.479 ± 0.002	0.318 ± 0.002^***^	−355.534	0.000
Alpha	0.384 ± 0.002	0.248 ± 0.001^***^	−342.028	0.000
Beta	0.289 ± 0.005	0.186 ± 0.003^***^	−107.552	0.000
Gamma	0.239 ± 0.009	0.153 ± 0.004^***^	−49.669	0.000

^***^p < 0.001.

### 3.3 Comparison of VMI-state data between groups

(1) Comparison of PSD

**Figure 3:**
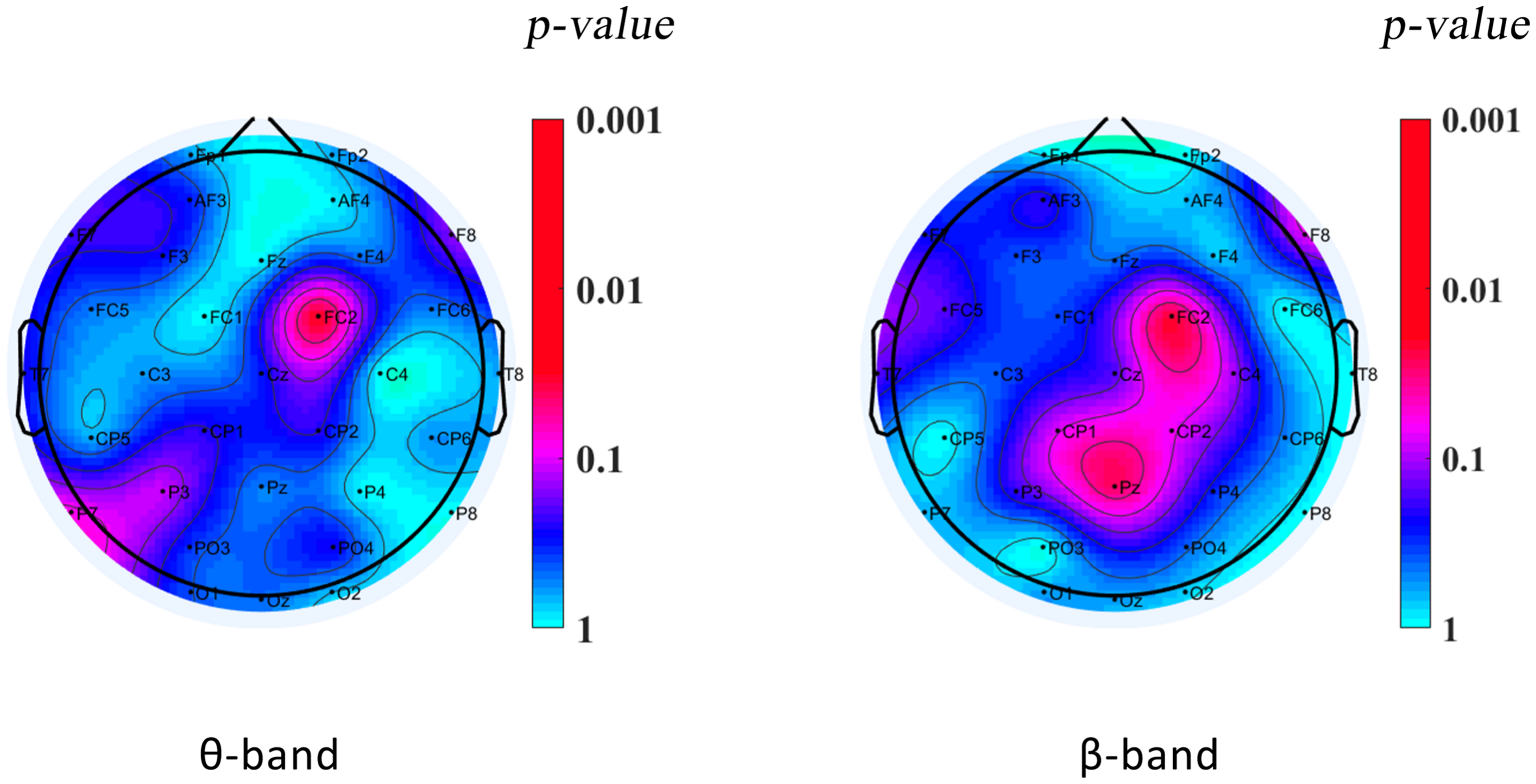
Plot showing PSD differences in the VMI state between DCD and TD boys.

During the VMI test, DCD boys’ relative power in the theta band at the right frontal-central zone junction (FC2) was significantly lower than that of TD boys (TD = 0.164 ± 0.088, DCD = 0.089 ± 0.521; *p* = 0.028), and their relative power in the beta band at the right frontal-central zone junction (FC2: TD = 0.072 ± 0.053, DCD = 0.006 ± 0.006; *p* = 0.000) and at the midline of the parietal zone (Pz: TD = 0.061 ± 0.052, DCD = 0.006 ± 0.004; *p* = 0.000) was significantly lower than that of TD boys. The power spectra of the other bands of the DCD boys did not significantly differ from those of the TD boys (*p* > 0.05). The results are shown in Table 4 and Figure 3.

(2) Comparison of SampEn

**Table 4:** Differences in the VMI-state PSD between DCD and TD boys.

Band	Channel	TD (*n* = 15)	DCD (*n* = 15)	*Z*	*p*
θ	FC2	0.164 ± 0.088	0.089 ± 0.521^*^	2.192	0.028
*β*	FC2	0.072 ± 0.053	0.006 ± 0.006^***^	3.780	0.000
	Pz	0.061 ± 0.052	0.006 ± 0.004^***^	3.628	0.000

^*^p < 0.05; ^***^p < 0.001.

In comparison, no statistically significant difference in the VMI-state EEG SampEn was found between the DCD boys and the TD boys (*p* > 0.05).

(3) Comparison of PLI values

**Figure 4:**
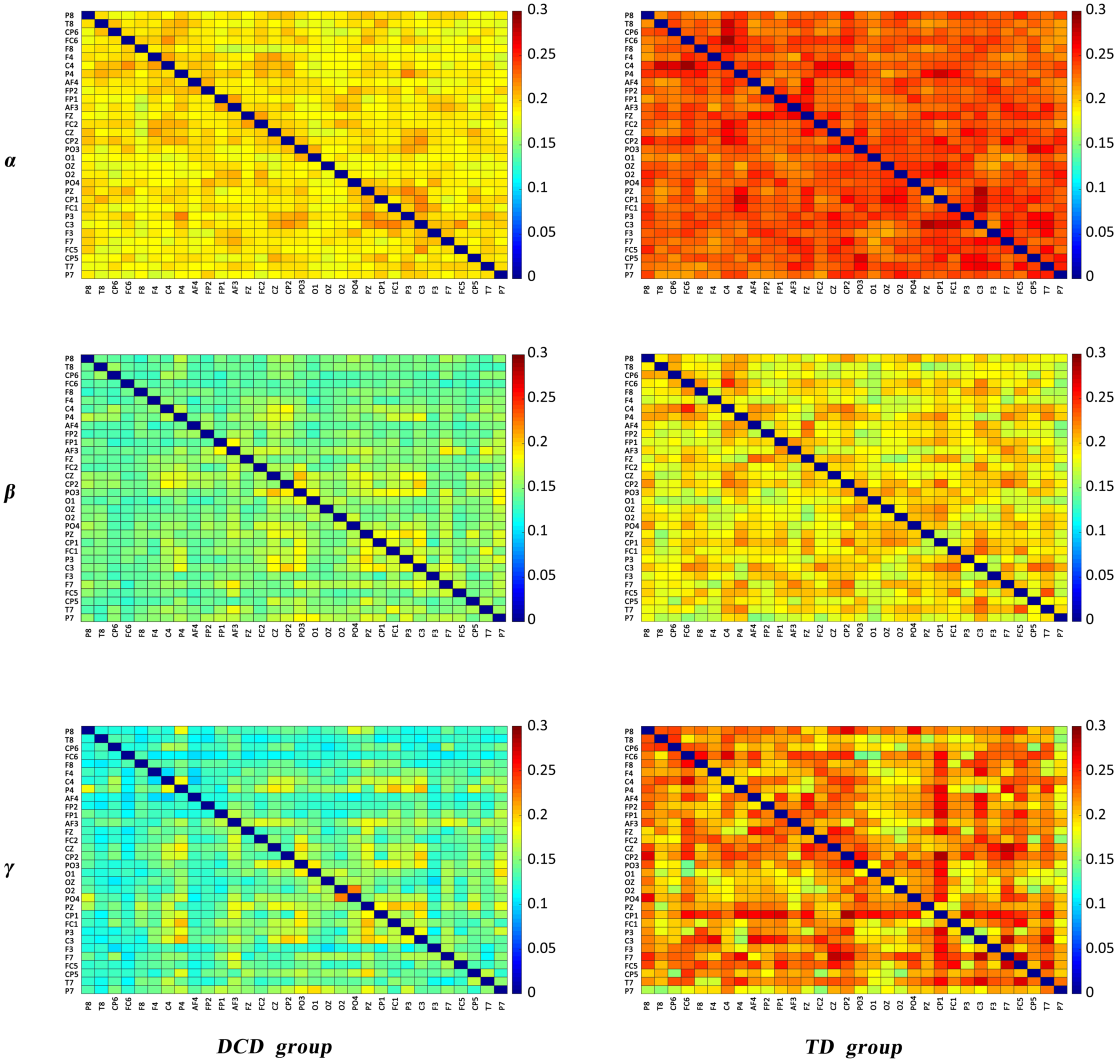
Matrix of PLI.

**Figure 5:**
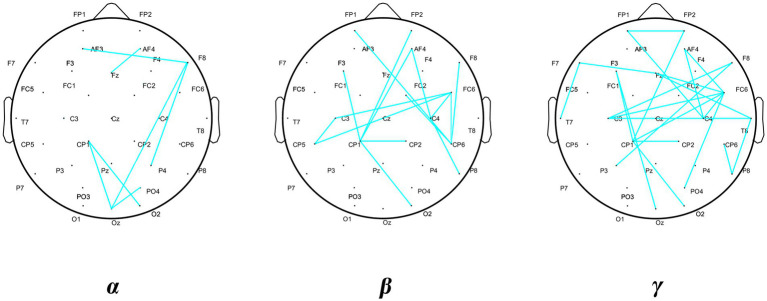
Differential distribution of PLI.

The results of the comparative analysis of the differences in PLI in the *δ*, *θ*, *α*, *β*, and *γ* bands between the DCD and TD boys revealed significant differences in PLI in the *α*, *β*, and *γ* bands between the groups, and the results of the comparison are shown in Figure 4. The differences in the figure are the mean differences in the PLI between specific channels for each group of subjects, and the strength of functional connectivity is indicated by color. There were significant differences in the PLI among the 7, 14, and 23 electrode pairs in the above three frequency bands, the results are shown in Figure 4 and Table 5. They are all connected by blue straight lines. For example: Fz to AF4 is connected by blue straight line, which represents a significant difference in the mean PLI from the frontal midline to the right frontal area between the two groups of children during VMI. The mean PLI values in the *α* (TD = 0.229 ± 0.004, DCD = 0.185 ± 0.003; *p* = 0.000), *β* (TD = 0.184 ± 0.006, DCD = 0.146 ± 0.007; *p* = 0.000), and *γ* (TD = 0.208 ± 0.011, DCD = 0.137 ± 0.011; *p* = 0.000) bands of the DCD boys were significantly lower than those of the TD boys as shown in Table 6. The statistical results suggested that the decrease in functional connectivity strength might be a potential influencing factor for DCD boys (see Figure 5).

(4) Comparison of functional connectivity lateralization

**Table 5:** Comparison of differences in PLI between DCD boys and TD boys.

Band	Brain region 1	Brain region 2	Electrode pairs	*p*
*α*	Left frontal region	Right frontal region	AF3-F8	0.016
	Right frontal region	Frontal zero	AF4-Fz	0.037
	Right frontal region	Occipital zero	F8-Oz	0.040
	Right frontal region	Right parietal region	F8-P4	0.044
	Left parietal region	Right occipital region	CP1-O2	0.021
	Left parietal region	Occipital zero	CP1-Oz	0.024
	Right occipital region	Occipital zero	PO4-Oz	0.043
*β*	Left frontal region	Right parietal region	FP1-CP6	0.036
	Right frontal region	Left parietal region	FP2-CP1	0.028
	Left frontal region	Left parietal region	F3-CP1	0.039
	Right frontal region	Left parietal region	AF4-CP1	0.047
	Right frontal region	Right central region	AF4-C4	0.049
	Right central region	Right parietal region	C4-P8	0.043
	Right central region	Left central region	FC6-C3	0.047
	Right central region	Right central region	FC6-C4	0.042
	Right central region	Left parietal region	FC6-CP5	0.043
	Left central region	Left parietal region	C3-CP5	0.027
	Left parietal region	Right parietal region	CP1-CP2	0.045
	Left parietal region	Right occipital region	CP1-O2	0.037
	Right central region	Right parietal region	FC2-CP6	0.036
	Right central region	Right parietal region	FC6-CP6	0.044
*γ*	Left frontal region	Right frontal region	FP1-FP2	0.034
	Left frontal region	Right central region	FP1-C4	0.049
	Right frontal region	Left parietal region	FP2-CP1	0.046
	Right frontal region	Right central region	AF4-C4	0.009
	Right frontal region	Right central region	AF4-FC6	0.047
	Left frontal region	Frontal zero	F7-Fz	0.048
	Left frontal region	Left temporal region	F7-T7	0.046
	Left frontal region	Left parietal region	F3-CP1	0.041
	Left frontal region	Occipital zero	F3-Oz	0.044
	Right frontal region	Left central region	F8-C3	0.040
*γ*	Right frontal region	Left parietal region	F8-P3	0.035
	Frontal zero	Right central region	Fz-FC6	0.013
	Frontal zero	Right central region	Fz-T8	0.036
	Right central region	Left central region	FC6-C3	0.026
	Right central region	Left parietal region	FC6-CP1	0.039
	Right central region	Right central region	FC6-C4	0.013
	Right central region	Right occipital region	FC6-PO4	0.039
	Right temporal region	Right parietal region	T8-CP6	0.025
	Left central region	Right temporal region	C3-T8	0.038
	Right temporal region	Right parietal region	T8-P8	0.045
	Right parietal region	Right parietal region	CP6-P8	0.011
	Left parietal region	Right occipital region	CP1-O2	0.020
	Left parietal region	Right parietal region	CP1-CP2	0.026

**Table 6:** Comparison of the mean PLI values of the two groups.

Band	TD (*n* = 15)	DCD (*n* = 15)	*t*	*p*
*α*	0.229 ± 0.004	0.185 ± 0.003^***^	45.852	0.000
*β*	0.184 ± 0.006	0.146 ± 0.007^***^	22.940	0.000
*γ*	0.208 ± 0.011	0.137 ± 0.011^***^	26.711	0.000

^***^p < 0.001.

Lateralization is an indicator of the difference in functional symmetry between the right and left cerebral hemispheres. Therefore, statistical processing was not performed on the functional connectivity between the two hemispheres or the central axis. According to the statistical results in Figure 4, the calculation and comparative analysis of the lateralization index of the PLI in the left and right hemispheres revealed that the right brain lateralization of the *β*-band functional connectivity in the frontal–central junction––central parietal junction (FC6-CP6: TD = 0.083 ± 0.143, DCD = −0.022 ± 0.006; *p* = 0.045) and frontal–prefrontal junction––central areas (AF4-C4: TD = 0.096 ± 0.158, DCD = −0.078 ± 0.103; *p* = 0.021) of the boys with DCD was significantly greater during the VMI task, whereas the control group showed a significant increase in the degree of left brain lateralization in the above brain areas. As shown in Table 7, the differences in brain lateralization may affect the performance of DCD boys.

**Table 7:** Results of the comparison of functional connectivity lateralization.

Band	Region	TD	DCD	*Z*	*p*
*α*	F8-P4	0.010 ± 0.108	0.022 ± 0.069	−0.189	0.850
*β*	C4-P8	−0.016 ± 0.111	−0.025 ± 0.141	0.416	0.678
	FC6-CP6	0.083 ± 0.143	−0.022 ± 0.006^*^	2.003	0.045
	F3-CP1	0.057 ± 0.193	−0.037 ± 0.142	0.340	0.734
	FC6-C4	0.087 ± 0.140	−0.079 ± 0.150	1.776	0.076
	AF4-C4	0.096 ± 0.158	−0.078 ± 0.103^*^	2.306	0.021
	C3-CP5	0.012 ± 0.121	−0.024 ± 0.165	0.340	0.734
*γ*	T8-P8	0.004 ± 0.257	−0.103 ± 0.195	0.869	0.385
	CP6-P8	0.120 ± 0.215	−0.019 ± 0.177	1.399	0.162
	FC6-C4	0.093 ± 0.178	−0.081 ± 0.225	1.474	0.141
	AF4-C4	0.006 ± 0.161	−0.155 ± 0.269	1.171	0.241
	FC6-PO4	0.061 ± 0.267	−0.053 ± 0.265	0.718	0.473
	F7-T7	0.049 ± 0.250	−0.194 ± 0.249	1.928	0.054
	F3-CP1	0.067 ± 0.239	−0.067 ± 0.145	0.945	0.345

Negative values indicate right brain lateralization, whereas positive values indicate left brain lateralization. ^*^p < 0.05.

## 4 Discussion

Electroencephalography, as a non-invasive medical detection technology, is widely used in neuroscience and cognitive neuroscience research due to its inclusion of a large amount of physiological and pathological information and ability to reflect the functional activity status of the brain. DCD is a common and well-recognized neurodevelopmental disorder, and its symptoms cannot be explained by other neurological conditions with motor impairments (20). The motor deficits typical of DCD children significantly impact the performance of daily activities that require movement, including dressing, handwriting (21). Therefore, this article aims to reveal the possible neural mechanisms underlying motor dysfunction in DCD children by examining their brain function during both resting and fine motor state. This study investigated the abnormal patterns of EEG power spectra, SampEn, PLI, and lateralization features in DCD boys.

### 4.1 Differences in resting-state EEG

In the resting state, no significant differences in brain activation were found between children with DCD and TD boys. Several studies have focused on EEG changes in children with DCD during motor or motor imagery tasks (11, 22, 23), but few studies have investigated their resting EEG characteristics. Keating et al. (14) suggested that the mirror neuron system (MNS) may lead to motor learning difficulties unique to DCD children. Therefore, they studied the resting state of MNS EEGs and reported no significant difference in the *μ* rhythm or alpha wave power between DCD children and TD children of the same age, which is consistent with the results of this study. This study not only did not find significant differences in alpha wave power between DCD boys and TD boys but also did not find any differences in EEG power spectra in other frequency bands, indicating that there are no abnormal changes in brain activation in DCD boys at rest. Synchronized neuronal firing is the basis of information processing in the brain. Synchronized neuronal firing occurs not only after exposure to sensory stimuli and during mental operations but also during spontaneous neural synchronization, which reflects the functional structure of the brain and serves as the basis for higher-order sensory and motor processes. The present study did not find any significant abnormal changes in resting-state brain activation levels in boys with DCD, which may be an important reason why they do not present specific mental developmental disorders. However, a recent study found that children with DCD exhibited significantly lower alpha power and higher delta power compared to the TD children (24). The DCD boys in the study showed significant ADHD symptoms, which may be an important reason for the different results of this study.

Under resting conditions, we found that DCD boys presented a significant decrease in PLI compared with TD boys. In a study of the EEGs of preterm infants with motor deficits growing to 9–10 years of age, a significant decrease in the mean PLI in the resting 8–10 Hz range was found in preterm infants (25). Although not identical to the findings of the present study, both studies reflect reduced levels of functional brain connectivity in DCD boys. Rinat et al. (26) used MRI to study resting-state functional connectivity in 8- to 12-year-old DCD children and reported that their sensory motor network functional connectivity was significantly lower than that of normal developing children of the same age. The changes in functional connectivity may reflect specific cognitive and perceptual states, as well as the ability to integrate information across brain regions. Therefore, the decrease in the resting PLI may be the biological basis for the occurrence of motor disorders in DCD boys.

### 4.2 Differences in EEG changes during the VMI task

(1) Differences in PSD

Human motor performance is related to theta wave activity (27, 28). The activation of theta waves may be related to the inhibitory effect on visual motor conversion, as frontal lobe executive function is required in the early stages of action to suppress established visual motor mapping (27). In addition, it has been shown that low-frequency oscillations play important roles in visual–motor planning and execution processes in the reinforcement of existing memories and the formation of new memories (28). Zion-Golumbic et al. (29) suggested that increased theta power in the frontal lobes reflects the utilization of long-term memory information during the processing of visual stimuli. Frontal-central theta rhythms are thought to be related to attentional control during movement (30). Therefore, it is speculated that the decrease in theta wave activation level in DCD boys may reflect their insufficient attention control level in the visual motor process. When performing VMI tasks, the activation of theta waves is related to cognitive activity levels such as memory, inhibition, and attention in the brain. Insufficient activation of theta waves in the right frontal central region (FC2) may be an important reason for poor motor performance in DCD boys due to cognitive impairment.

Beta waves are the EEG band associated with sensorimotor processes (31). Tan et al. (32) showed that increased beta-band activity may reflect neural processes involved in assessing actual and expected exercise outcomes, with increased beta-band activation in trials with small errors and decreased beta-band activation in trials with large errors. Kamiński et al. (33) demonstrated that increased activity within the beta wave may be a vector of attentional arousal in humans. There is a positive correlation between parietal beta-wave activity and accuracy in a visual vigilance task, which may be an indication of the gain of enhanced feedback loops during subsequent stages of visual information processing (34). A study reported that DCD children had reduced beta wave activity in their brain motor areas when completing finger movement tasks (24). This is consistent with the results of this study.

In summary, the decreased level of beta wave activation in the parietal region of the DCD boys in the present study during the VMI task may reflect their poor level of visual attention during the visual–motor integration task and their weak executive feedback ability after visual information is processed, thus demonstrating deficiencies in hand–eye coordination.

(2) Differences in PLI

The Motor skills learning requires the involvement and integration of several cortical and subcortical areas. It has been found that high levels of motor skill are associated with stronger functional brain connectivity. It has been reported that soccer players at national level 2 and above have significantly enhanced brain functional connectivity in the *α* and *β* frequency bands in the resting state, compared with physical education students without motor level (35). The resting-state frontal–parietal network and the default mode network were found to be more activated in basketball players after long-term training. Similar studies have found greater activation of the resting-state frontal–parietal network, the default-mode network, in basketball players who have undergone long-term training (36). Based on the paradigm linking increased connectivity between brain regions to interregional cooperative processes, EEG studies of hand movement tasks have shown that functional connectivity in different brain regions is also enhanced during task performance. It has been found that the level of functional connectivity in all frequency bands was elevated in subjects during the completion of hand movements compared to resting, and that deficits in B-band functional connectivity were demonstrated in patients with focal hand dystonia during the performance of a simple finger-tapping task (37).

In the present study, the strength of brain functional connectivity in the *α*, *β*, and *γ* frequency ranges was significantly lower in the DCD boys than in the TD boys during the VMI task, suggesting that the functional connectivity between different brain regions in the brains of the DCD boys was lower than that of the control group during the execution of movements involving hand–eye coordination, which may have a negative impact on their motor performance.

However, the findings of Pranjić et al. (38) are inconsistent with those of the present study. Children with DCD exhibited reduced interhemispheric connectivity during program adjustment and showed similar neural patterns regardless of task constraints compared with their TD peers. The reason for the different results may be the different time periods in which the data were analyzed. Pranjić et al. (38) reported that the movement planning stage was characterized, whereas the results of the present study characterized the entire movement process.

On the basis of the findings of elevated resting-state brain functional connectivity in high-level athletes and reduced brain functional connectivity during hand movements in patients with focal hand dystonia, the present study suggests that boys with DCD have reduced brain functional connectivity at rest and during movements compared with TD boys of the same age.

(3) Differences in the lateralization of functional brain connectivity

Lateralization is one of the properties of the brain that enables efficiency. Multitasking functional activity, with the left hemisphere playing a decisive role in verbal functioning, logical thinking, analytical ability, and computation, and the right hemisphere having a clear advantage in spatial functioning, shape recognition, fine arts, integrative ability, and transient visual memory (39). Lateralization of brain function is an important organizational feature of the motor system (40), which is also necessary for various coordinated movements (41). In the present study, the *β*-band functional connectivity at the frontal central junction–central parietal junction and frontal prefrontal junction–central junction of normal-developing boys was found to be left lateralized in the graphic copying task, whereas it was right lateralized in boys with DCD. The central region is a brain region classified by the international standard for the location of EEG electrodes, and its corresponding brain region is the motor region of the brain. A study of “mirror movement” in humans revealed that lateralized activation of the brain is realized by contralateral inhibition (41). The left hemisphere of the brain is dominant in the ability to plan and coordinate motor movements effectively. The left hemisphere of the brain is dominant in the ability to effectively plan and coordinate motor movements (42). The dominance of the left hemisphere in the human motor cortex in right-handed subjects has been reported (43). It was hypothesized that the abnormal lateralization of functional connectivity in DCD boys during VI exercise reflected their deficits in motor planning and coordination. Hauk and Pulvermiiller reported that the brains of subjects stimulated with a hand motor vocabulary presented a pattern of left-lateralized activation (44). Therefore, the right lateralization of perimotor connectivity in the DCD boy in the present study may be an important manifestation of his cortical motor area function. Therefore, the right lateralization of functional connectivity in the periphery of the motor area in the boys with DCD in the present study may be an important manifestation of the abnormal function of the motor area of their cerebral cortex.

## 5 Conclusion

In this study, boys with DCD demonstrated significantly lower functional brain connectivity (measured by phase lag index, PLI) across all frequency bands during rest, despite no significant abnormalities in EEG relative power or signal complexity (SampEn). During visual–motor integration (VMI) tasks, DCD boys showed reduced theta and beta power in specific frontal-central and parietal regions, along with significantly reduced PLI in *α*, *β*, and *γ* bands. Furthermore, abnormal right-hemispheric lateralization in *β*-band connectivity was observed in motor-related regions. These findings indicate that altered functional connectivity and hemispheric asymmetry, rather than general cortical activation or complexity, may be associated with motor coordination difficulties in boys with DCD. However, given the limited sample size and the cross-sectional design, these findings should be interpreted with caution and warrant further investigation.

## Data Availability

The raw data supporting the conclusions of this article will be made available by the authors, without undue reservation.
